# Suppression of the antitumoral activity of natural killer cells under indirect coculture with cancer-associated fibroblasts in a pancreatic TIME-on-chip model

**DOI:** 10.1186/s12935-023-03064-9

**Published:** 2023-09-27

**Authors:** Hyun-Ah Kim, Hyunsoo Kim, Min-Kyung Nam, Jong Kook Park, Moo-Yeal Lee, Seok Chung, Kyung-Mi Lee, Hyo-Jeong Kuh

**Affiliations:** 1 Department of Biomedicine & Health Sciences, Graduate School, The Catholic University of Korea, Seoul, Republic of Korea; 2 School of Mechanical Engineering, College of Engineering, Korea University, Seoul, Republic of Korea; 3 Cancer Evolution Research Center, College of Medicine, The Catholic University of Korea, Seoul, Republic of Korea; 4 Department of Biomedical Science and Research Institute for Bioscience & Biotechnology, Hallym University, Chuncheon, 24252 Republic of Korea; 5 Department of Biomedical Engineering, University of North Texas, 3940 North Elm Street, Denton, TX 76207 USA; 6 Department of Biochemistry and Molecular Biology, Korea University College of Medicine, Seoul, Republic of Korea; 7 Department of Medical Life Sciences, College of Medicine, The Catholic University of Korea, 222 Banpo-daero, Seocho-ku, Seoul, 06591 Republic of Korea

**Keywords:** Natural killer cell, Pancreatic stellate cell, Pancreatic ductal adenocarcinoma, Tumor spheroid, Tumor immune microenvironment, 3D coculture system, TIME-on-chip model

## Abstract

**Background:**

Recently, natural killer (NK) cells emerged as a treatment option for various solid tumors. However, the immunosuppressive tumor immune microenvironment (TIME) can reduce the cytotoxic ability of NK cells in pancreatic ductal adenocarcinoma. Cancer-associated fibroblasts within the tumor stroma can suppress immune surveillance by dysregulating factors involved in the cellular activity of NK cells. Herein, the effect of activated pancreatic stellate cells (aPSCs) on NK cell-mediated anticancer efficacy under three-dimensional (3D) coculture conditions was investigated.

**Methods:**

3D cocultures of PANC-1 tumor spheroids (TSs) with aPSCs and NK-92 cells in a collagen matrix were optimized to identify the occurring cellular interactions and differential cytokine profiles in conditioned media using microchannel chips. PANC-1 TSs and aPSCs were indirectly cocultured, whereas NK-92 cells were allowed to infiltrate the TS channel using convective medium flow.

**Results:**

Coculture with aPSCs promoted PANC-1 TSs growth and suppressed the antitumor cytotoxic effects of NK-92 cells. Mutual inhibition of cellular activity without compromising migration ability was observed between aPSCs and NK-92 cells. Moreover, the reduced killing activity of NK-92 cells was found to be related with reduced granzyme B expression in NK cells.

**Conclusions:**

Herein, a novel TIME-on-chip model based on the coculture of PANC-1 TSs, aPSCs, and NK-92 cells was described. This model may be useful for studying the detailed mechanisms underlying NK cells dysregulation and for exploring future therapeutic interventions to restore NK cell activity in the tumor microenvironment.

**Supplementary Information:**

The online version contains supplementary material available at 10.1186/s12935-023-03064-9.

## Background

Pancreatic ductal adenocarcinoma (PDAC) is one of the most lethal malignancies worldwide, owing to its high mortality rate and poor prognosis [1]. Recently, immunotherapy was proposed as a potential treatment option for PDAC cases that do not respond to chemotherapy [2]. Although T cells have been comprehensively studied for the development of cancer immunotherapies [3], natural killer (NK) cells are another type of tumor-infiltrating lymphocytes that are known to play a critical role in the antitumor immune response [4]. Owing to their innate ability to recognize and rapidly eliminate tumor cells and for contributing to the production of cytokines that modulate adaptive immune responses, NK cell-based immunotherapies have emerged as efficacious anticancer treatments and are currently the focus of clinical investigations. Despite the current challenges regarding the source of NK cells and methods for their stimulation, NK-based therapeutic strategies, such as chimeric antigen receptor (CAR)-transduced NKs, are being pioneered as potential anticancer treatments [5].

The tumor immune microenvironment (TIME) refers to immune cell populations, including myeloid cells, lymphocytes, and other innate immune cells, that infiltrate the tumor microenvironment [3]. PDAC has been characterized as a desmoplastic tumor, considered an immunologically ‘cold’ tumor, in which cytotoxic lymphocytes are excluded from the tumor parenchyma and are localized along the invasive margin of the tumor mass or are caught in fibrotic nests [6]. In particular, NK cells from patients with PDAC have reduced tumor infiltration capacity owing to their low chemokine receptor expression [7]. NK cell dysfunction in PDAC has been reported to occur through the reduced production of cytotoxic granule components, granzyme B, and perforin, as well as by the reduced expression of active receptors, including NKG2D, NKp46, and NKp30, as compared with healthy individuals [8–11]. Indeed, previous reports have described molecular mechanisms underlying the phenotypic changes in NK cells towards a tumor-promoting subtype [12, 13]. Nevertheless, further studies are necessary to better understand the immunosuppressive microenvironment of PDAC.

Tumor microenvironmental factors include cellular components, extracellular matrix (ECM), and abundant soluble signaling molecules [14]. Cancer-associated fibroblasts (CAFs) are one of the most important and abundant cell types within the tumor microenvironment and are responsible for tumor-promoting functions, including the production of growth factors, promoting ECM remodeling, and by modulating metabolism and angiogenesis [15]. CAFs are major players in ECM protein deposition within the tumor stroma, which will create physical barriers to therapeutic agents and immune cells, including NK cells [16, 17]. Noteworthily, pancreatic stellate cells (PSCs) can transform into activated PSC (aPSC), a type of myofibroblast-like cells that can act as CAFs. These cells are key regulators that evade immune surveillance and consequently contribute to tumor progression in patients with PDAC [18, 19]. aPSCs from the pancreatic tumor stroma have been shown to exert a negative regulatory effect on NK cells, thereby being completely different from quiescent normal PSCs in peritumoral tissues [20]. Moreover, the protumoral function of NetG1-expressing CAFs has been attributed to the inhibition of the cancer cell killing ability of NK cells [21]. Due to these characteristics, aPSCs have recently been identified as a cause of immunosuppression in pancreatic cancer.

The organized three-dimensional (3D) structure of the tumor microenvironment has been recognized as an important attribute; hence, in vitro tumor models have been developed using several platforms to better mimic 3D cell-to-cell and cell-to-ECM interactions [22], but also to better understand the microenvironmental factors affecting the immune cell function against cancer [23], elucidate the regulation of NK cell migration [24], and screen drug combinations for NK cell-mediated cytotoxicity [25]. Microfluidic chip-based 3D models have also been used to study the role of microenvironmental stress and the effects of antibody conjugates on NK cell function [26, 27]. In the present study, a 3D in vitro TIME-on-chip model of human pancreatic tumors was established using microfluidic chips to investigate the effects of aPSCs on NK cell-mediated cancer cell death. A 3D coculture system composed of cancer cells growing as tumor spheroids (TS), aPSCs, and NK cells was developed, and the validity of the model was demonstrated by mutual phenotypic changes in the cells included in the system.

## Methods

### Cell culture

PANC-1, a human pancreatic cancer cell line, and NK-92, a human NK cell line, were purchased from American Type Culture Collection (Manassas, VA, USA). PANC-1 cells were cultured in high-glucose DMEM (Hyclone, Logan, UT, USA) supplemented with 10% fetal bovine serum (FBS; Welgene, Daegu, Korea), 100 µg/mL streptomycin, 100 units/mL penicillin, and 250 ng/mL amphotericin B (Welgene). NK-92 cells were cultured in minimum essential media alpha (α-MEM; 12561056, Gibco, BRL, Life technologies CO. USA) supplemented with 20% FBS and interleukin (IL)-2 (200-02; Peprotech, Cranbury, NJ, USA). NK-92 cells were sub-cultured every other day in medium containing IL-2 at 300 units/mL. Human pancreatic stellate cells (PSCs), purchased from ScienCell (HPaSteC, #3830, Carlsbad, CA, USA) were cultured in Stellate Cell Medium (SteCM; #5301, ScienCell, Carlsbad, CA, USA) supplemented with 2% FBS, growth supplement, and 1% antibiotic solution, as recommended. For differentiating naïve PSCs, cells were activated in high-glucose DMEM supplemented with 5% fetal bovine serum, at least 72 h prior to use in experiments. Green fluorescent protein (GFP)-expressing PANC-1 cells were generated as described previously [28]. Briefly, the pEGFP-N1 plasmid was transfected into PANC-1 cells using FuGENE HD reagent (Promega, Madison, WI, USA) and a clonal cell line was selected using G418 (0.4 mg/mL). The cells were maintained at 37 °C in a humidified 5% CO_2_ atmosphere.

### Fabrication of a polydimethylsiloxane (PDMS) microchannel chip

The microchannel chips consisted of two cell channels and three media channels. An SU-8 patterned master was custom-made (AMED, Seoul, Korea) using photolithography. The microchannel chips were fabricated using soft lithography according to a previously reported protocol [29, 30]. The widths of the cell-loading and media channels were 700 and 1,000 μm, respectively, and a height of 250 μm. Briefly, a PDMS mixture was prepared by mixing the curing agent and PDMS prepolymer (SYLGARD 184 Silicone Elastomer Kit; Dow Chemical, Midland, MI, USA) at a weight ratio of 1:10. The mixture was then poured onto a patterned master, degassed for 20 min in a vacuum desiccator, and cured in a drying oven at 60 °C for 3 h. After detaching the PDMS mold from the master, cell loading ports and media reservoirs were formed using a 1 mm needle and a 4 mm biopsy punch, respectively, before the mold was attached to a glass coverslip using oxygen plasma treatment (CUTE; Femto Science, Seoul, Korea). All channels in the chips were rinsed with a polydopamine solution (2 mg/mL) and allowed to dry for 2 h to coat the channel surface. Following washing with deionized water twice, the microchannel chip was dried in an oven at 60 °C.

### 3D culture on a microchannel chip

Type I collagen solution was prepared as a cell suspending medium at 1.0–1.5 mg/mL by mixing phenol red, 0.5 N NaOH, rat tail tendon type I collagen (354236; Corning, Bedford, MA, USA), and sterilized distilled water. PANC-1 and PSCs were suspended at densities of 5 × 10^5^ and 1 × 10^6^ cells/mL, respectively. The cells were then loaded into the chip by injecting 4.7 µL of the cell-collagen mixture in each designated channel, placing 2.3 × 10^3^ cells/channel of PANC-1 cells and 4.7 × 10^3^ cells/channel of PSCs in the effective area of each microchannel. After gelation in a cell culture incubator for 35 min, the media channels were infused with 100 µL of medium and cultured for up to 5 days in a 5% CO_2_ incubator with daily media changes.

**Fig. 1:**
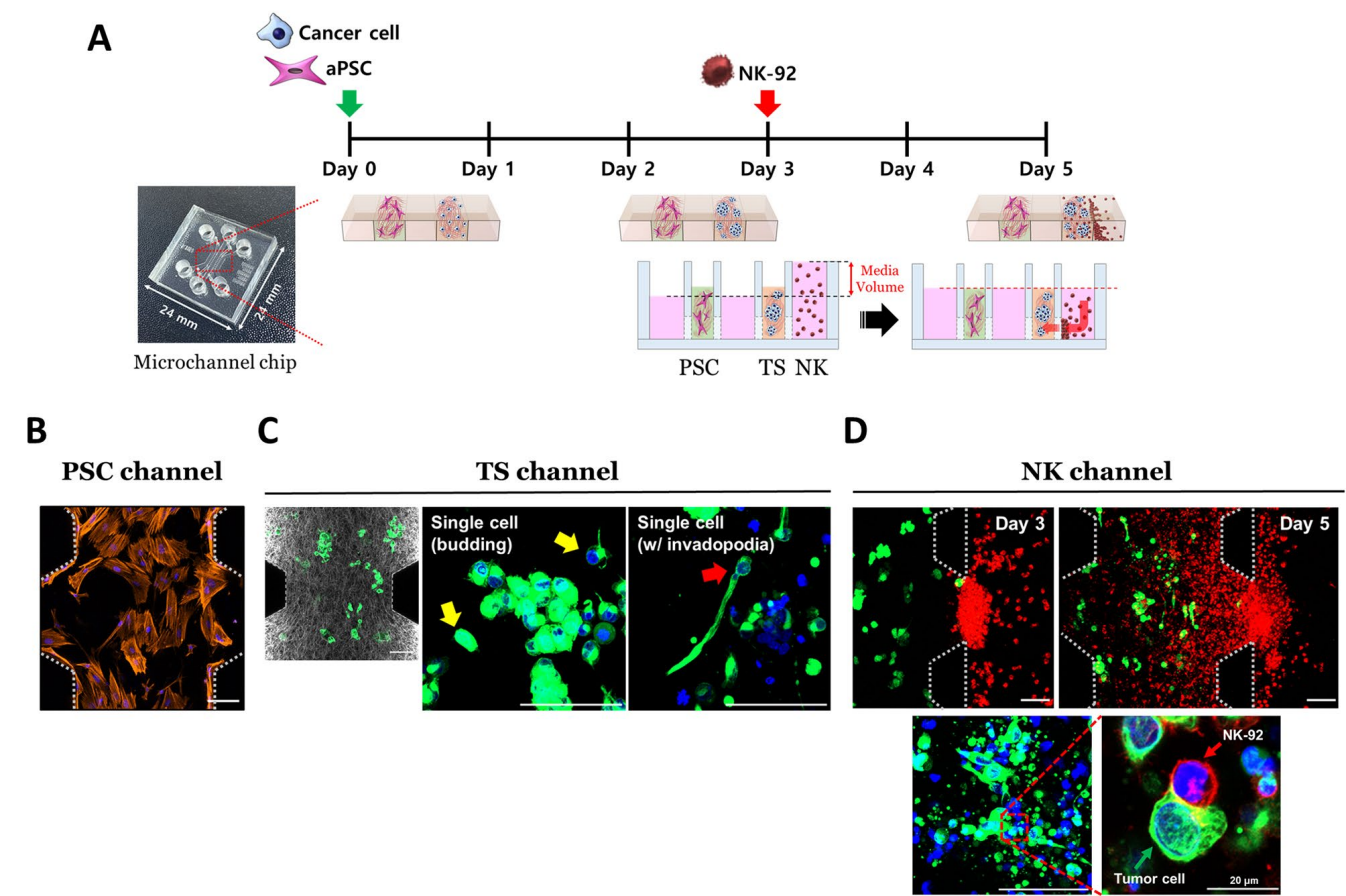
Schematic illustration of 3D coculture of PANC-1 TS, aPSCs, and NK-92 cells in the TIME-on-Chip model using microchannel chips. (**A**) PANC-1 cells and aPSCs were loaded 3 days before loading NK cells. When loading NK-92 cells, different volumes of media were used to generate a hydraulic pressure, initiating the convectional movement of the cells. Images of the respective microchannel containing aPSCs (**B**), PANC-1 TSs (**C**), and NK-92 cells (**D**) after 5 days of culture. Orange (F-actin): aPSCs, blue (DAPI): nuclei, green (cytokeratin-19 or GFP): PANC-1 TS, and red (PKH26): NK-92 cells. Experiments were conducted at 1.2 mg/mL collagen concentration. Tumor cells and aPSCs were loaded in the designated channel as embedded in the collagen matrix and images were collected after 2 days, allowing the interaction between NK-92 cells and PANC-1 TSs. Three fields were observed from each microchannel chip for analysis. Scale bar: 100 μm. 3D: three dimensional; aPSC: activated pancreatic stellate cell; GFP: green fluorescent protein; TIME: tumor immune microenvironment; TS: tumor spheroid

NK-92 cells loading was performed by replacing the culture media in the media channel adjacent to the tumor channel with 100 µL of 2.5 × 10^5^ cells/mL of NK-92 cell suspension after 3 days of coculture of PANC-1 and aPSCs. Then, the remaining media channels were filled with only 60 µL of the culture medium to generate convective media flow by hydraulic pressure. Fluid movement occurred within 24 h until the media volume equalized in each media reservoir and the NK-92 cells were stacked toward the neighboring collagen channels. The infiltration and migration of NK-92 cells into the collagen channel were allowed in the presence or absence of TSs for 2 days without changing the media (Fig. 1A).

### Cell tracker and immunofluorescence staining

NK-92 cells were tracked using a fluorescent cell tracker dye (PKH26, Red Fluorescent Cell Linker Kit; Sigma-Aldrich, Saint Louis, MO, USA) according to the manufacturer’s instructions. Dead cells were visualized using propidium iodide (PI) staining (BDA-1000; BIOMAX, Seoul, Korea). For immunofluorescence staining, the cells were first fixed using 4% paraformaldehyde (PFA) or methanol for 20 min and non-specific binding was blocked using 10% normal goat serum for 2 h at room temperature. The samples were then incubated with primary antibodies against α-SMA (1:150, ab5694, Abcam, Cambridge, UK), CD56 (1:150, MA5-11563, Invitrogen), collagen type 1 (1:200, ab6308, Abcam), cytokeratin 19 (1:300, ab52625, Abcam), FAP-α (1:200, ab28244, Abcam), Fibronectin (1:200, ab2413, Abcam), granzyme B (1:100, MA1-80734, Invitrogen), IL-6 (1:200, ab214429, Abcam), NKp46 (1:50, MAB1850, R&D system), perforin (1:200, 14-9994-82, Invitrogen), and TGF-β1 (1:200, ab92486, Abcam) overnight or for 2 days at 4 °C. The samples were washed with phosphate-buffered saline and stained with anti-rabbit or anti-mouse secondary antibodies conjugated with Alexa Fluor 594 (A11012) or 488 (A11008 and A11029). Rhodamine phalloidin (1:500, R415, Invitrogen, Carlsbad, CA, USA) and DAPI (1:1000, D9564, Sigma Aldrich, St. Louis, MO, USA) were used to stain F-actin and nuclei, respectively.

### Image acquisition and analysis

Fluorescence images were acquired using a confocal microscope (LSM 800 W/Airyscan; Carl Zeiss, Oberkochen, Germany). The analysis of an entire channel was analyzed using tile imaging techniques, covering at least 80% of the effective area. Optical sections were acquired at 5 μm intervals at 50× and 100×, 4 μm at 200×, and 1.5 μm at 400× magnifications to stack into z-projection images. 3D reconstruction was performed using ZEN software (Carl Zeiss). Fluorescence intensity was determined using the ZEN software, and cellular marker expression in aPSCs and NK-92 cells was normalized to DAPI and CD56 signals, respectively. Image analysis was performed using ImageJ software (National Institutes of Health, Bethesda, MD, USA) to determine the number and morphology of cells and TSs, and the structure of the ECM. The thickness of the ECM fibers, including collagen type 1 and fibronectin, was analyzed using the BoneJ plugin in ImageJ. The diameter of the TSs and NK cells was calculated as 2 × (area/π)^1/2^ assuming a spherical shape. Cells with a diameter of 10–20 μm were considered single cells.

### Secretome analysis in the conditioned media

Differential levels of cytokines released in conditioned media were analyzed using the Human Cytokine Array C1 (RayBiotech, Norcross, GA, USA) according to the manufacturer’s instructions. Briefly, human cytokine antibody membranes were incubated in blocking buffer for 30 min and then incubated with 1.5 mL of conditioned media samples at 4 °C overnight. After washing with buffer, the membranes were incubated in a diluted biotinylated antibody cocktail for 2 h at room temperature. The membranes were then incubated with horseradish peroxidase-streptavidin for 2 h at room temperature and subjected to chemiluminescence reactions and imaging. Densitometry was performed to quantify spot intensities using GeneTools software (Syngene, Cambridge, UK).

### Statistical analysis

All data are expressed as the mean ± standard deviation (SD) of three independent experiments. Statistical significance was determined using Student’s *t*-test or one-way analysis of variance followed by Tukey’s post-hoc test using Microsoft Excel 2010 (Microsoft Corporation, Redmond, WA, USA) and GraphPad Prism 9.0 (GraphPad Software, Boston, MA, USA). Statistical significance was set at *p* < 0.05.

## Results

### Development of a 3D TIME model using a microchannel chip

A triple coculture of PANC-1 TSs, aPSCs, and NK-92 cells was optimized in microchannel chips to evaluate tumor growth and NK cell infiltration. In particular, NK cell-mediated cancer cell killing was demonstrated under 3D conditions in vitro (Fig. 1A). The activated phenotype of aPSCs was well maintained in the collagen matrix, as confirmed by the flat shape of the cells along with the abundance of actin stress fibers (Fig. 1B). Furthermore, a 2 to 5-fold higher expression of α-SMA (α-smooth muscle actin) and FAP-α (fibroblast activation protein-α) was observed in aPSCs compared to naïve PSCs (data not shown). PANC-1 cancer cells were grown in a collagen matrix to form TSs with a mean diameter of 41.7 μm after three days of culture before loading the NK cells. Particularly, the PANC-1 TSs were distributed in varying sizes and shapes in the 3D space of the tumor channel. The invasive morphology of PANC-1 TSs was observed by membrane protrusion and tumor budding as single cells disseminated in the ECM (Fig. 1C). After successful loading of NK-92 cells using convective flow of culture media on day three (Fig. 1A), approximately 50% of these cells infiltrated into the collagen matrix channel, of which 7% were found in the distal half of the region by day five (Fig. 1D). Cellular contact between PANC-1 cells and infiltrating NK-92 cells was observed in the tumor channel, indicating that the anticancer response of NK-92 cells was well-preserved in the culture (Fig. 1D). Overall, these results demonstrate the successful modeling of the TIME for examining NK cell-mediated anticancer effects.

### Effect of matrix collagen concentration on NK cell infiltration

**Fig. 2:**
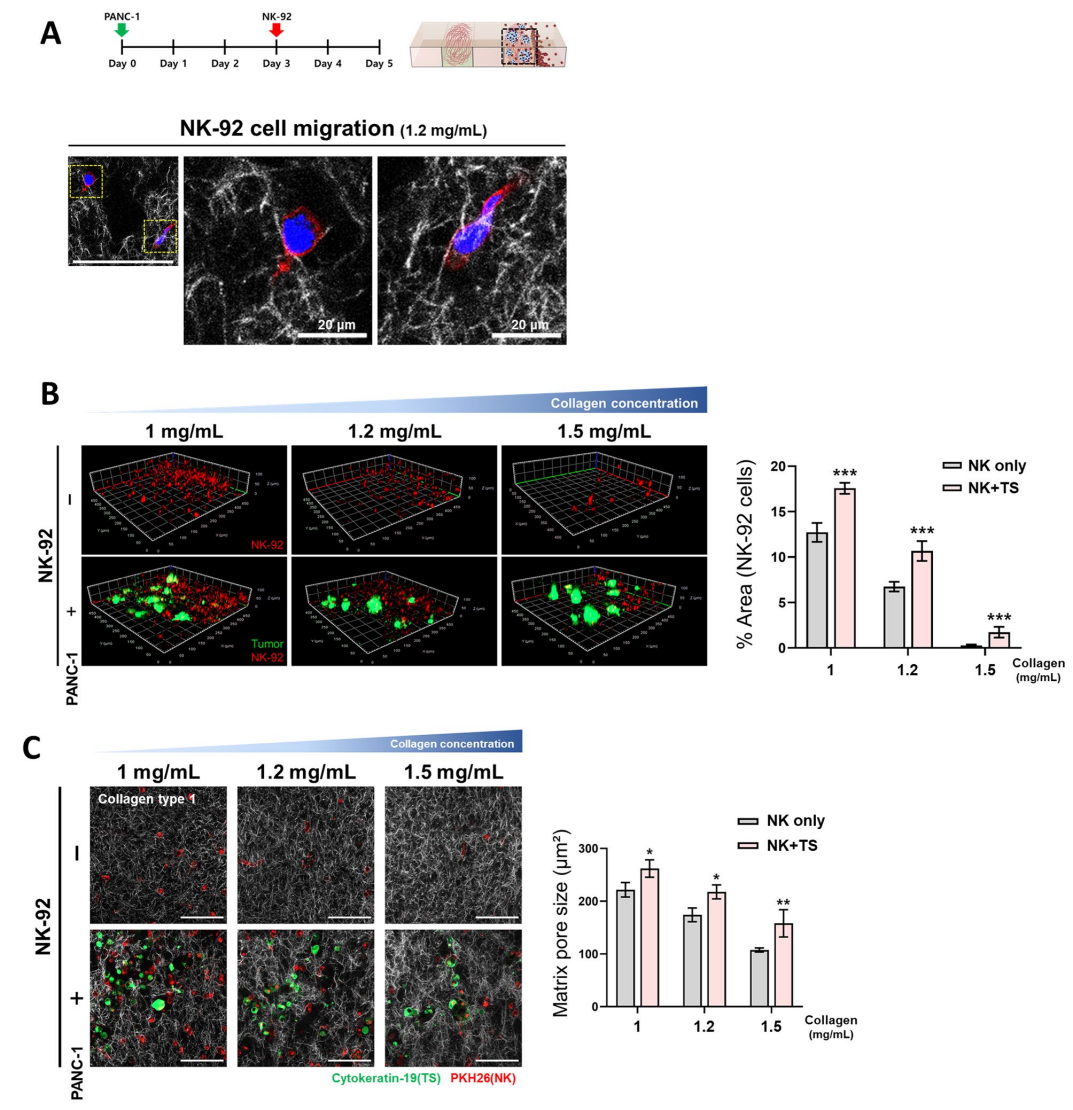
Changes in NK cell penetration according to the matrix collagen concentration. (**A**) Migration of NK-92 cells in a collagen matrix (1.2 mg/mL) when cocultured with PANC-1 TSs. (**B**) Levels of NK-92 cell infiltration in the tumor channel and (**C**) matrix pore size at different concentrations of matrix collagen with or without PANC-1 TSs coculture. Tumor cells were loaded in the designated channel as embedded in the collagen matrix and images were taken after 2 days of NK cells loading, thereby allowing the interaction between PANC-1 TSs and NK-92 cells. Visualization was performed using GFP- or cytokeratin-19-labelled PANC-1 cells and NK-92 cells were stained with the PKH26 tracker. Collagen type 1 fibers are shown with white pseudo color and overlaid with PANC-1 TS (green) and NK-92 (red). Scale bar: 100 μm. Minimum of 10 regions of interest were selected from three fields of each microchannel chip. Data represent mean (± SD) values of three independent experiments. *p < 0.05, **p < 0.01, ***p < 0.005

NK-92 cells penetration into the matrix was examined at varying collagen concentrations (1–1.5 mg/mL), either in the presence or absence of PANC-1 TS, 48 h after loading. These exhibited amoeboid-like migration patterns through the collagen matrix, as determined by morphological changes (Fig. 2A). The degree of NK-92 cell infiltration was determined as the percentage area occupied by the fluorescent signal from the PKH26 tracker-loaded NK cells. NK-92 cell distribution decreased from 13 to 1% and 18 to4% with increasing collagen concentration from 1 to 1.5 mg/mL, in the absence and presence of PANC-1 TSs, respectively (Fig. 2B). A significantly higher infiltration of NK cells was observed, with 1.38- and 1.6-fold increases at 1 and 1.2 mg/mL collagen concentrations, respectively, in the presence of TS compared with that in cell-free matrix (Fig. 2B). The pore sizes in the collagen matrix also showed concentration dependence; the matrix pore size (cross-sectional area) decreased from 222 to 108 µm^2^ and from 262 to 158 µm^2^ with increasing concentrations of collagen in the absence and presence of PANC-1 TSs, respectively (Fig. 2C). The pore size of the collagen fiber network increased by 1.18- to 1.46-fold in the presence of PANC-1 TSs. The minimal (1–4%) infiltration of NK-92 cells may be attributed to the significant resistance from pore sizes of less than 160 µm^2^ (at 1.5 mg/mL collagen) when considering 227 µm^2^ of the cross-sectional area of NK-92 cells (cell diameter of 17 μm in suspension culture or collagen matrix). Based on these results, matrix with 1.2 mg/mL type I collagen was used in subsequent experiments.

With their amoeboid-like migration, NK-92 cells appeared to migrate through the collagen network without causing matrix remodeling, which was further supported by the lack of differences in pore size measured in the collagen matrix with or without NK-92 cells (Fig. 2C vs. Additional file 1: Fig. S1). Note that the area with low collagen density represents the region with matrix remodeling induced by PANC-1 TSs, but not by NK-92 cells.

### Changes in cytokine secretion under coculture conditions

**Fig. 3:**
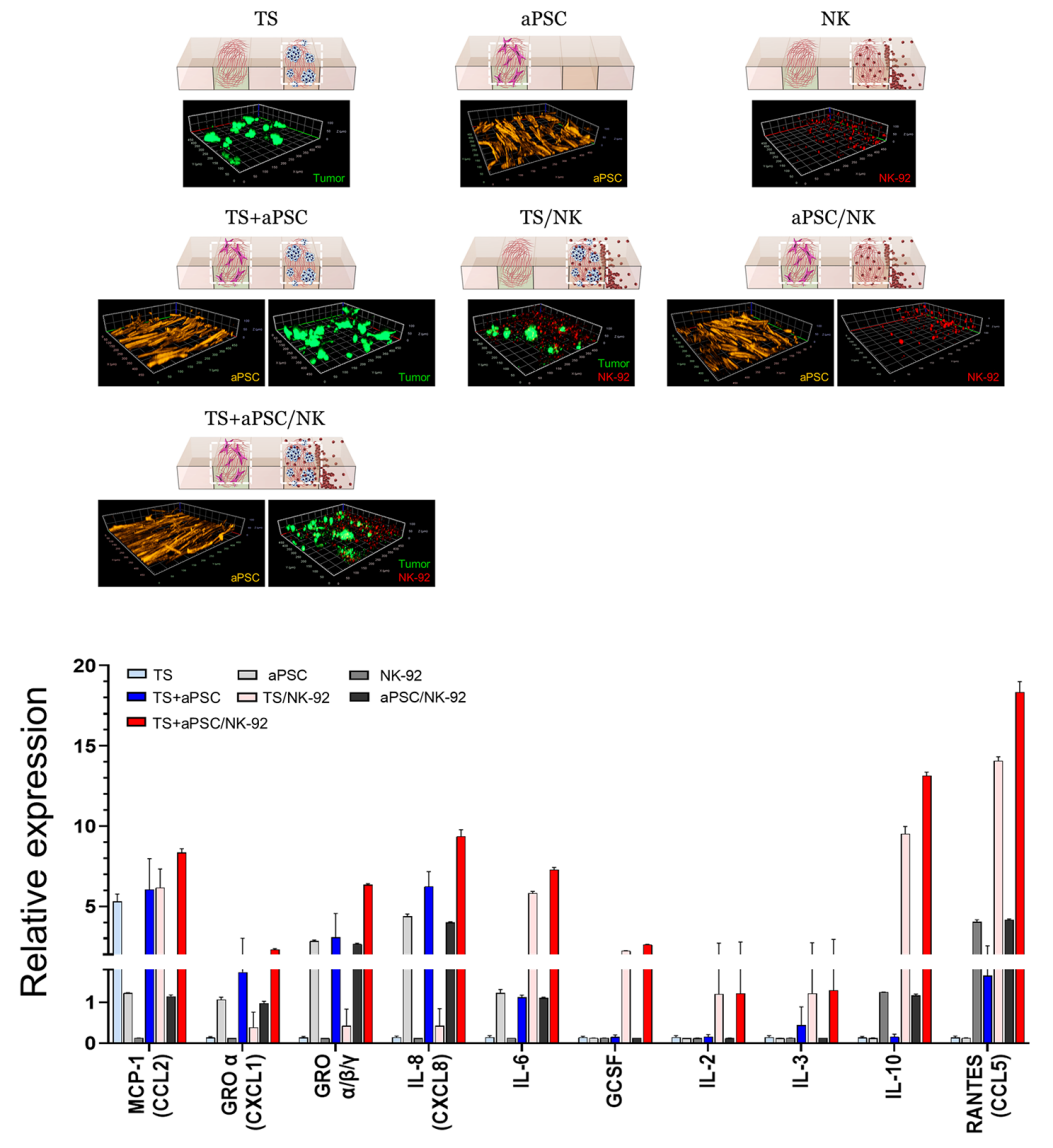
Secretome analysis of conditioned media in PANC-1 TS cocultured with aPSCs and NK cells using microchannel chips. 3D images of cell channels of each culture group obtained by using GFP-labelled PANC-1 cells, PKH26 tracker-loaded NK-92 cells, and aPSCs stained for F-actin (orange). Only 10 proteins showed significant levels of expression in PANC-1 TSs, aPSCs, and NK cells cultured alone or under coculture conditions. Data represent mean (± SD) values of three independent experiments

To validate the cell-cell interactions occurring under the TSs, NK-92 cells, and aPSCs coculture system, cytokines in the conditioned media were analyzed. Among 23 analyzed cytokines, only 10 factors were found to be differently secreted to the medium between the coculture groups (Fig. 3 and Additional file 2: Fig. S2**A**). MCP-1 (Monocyte chemoattractant protein-1) levels were significantly higher in the experimental groups containing TSs, and to a lesser extent, in the groups containing only aPSCs. GROα/β/γ, IL-8, and IL-6 showed a significant expression in culture groups containing aPSCs, suggesting that aPSCs are major cells secreting these factors. IL-6 and three other factors, namely G-CSF (granulocyte-colony stimulating factor), IL-2, and IL-3, showed a significant expression in coculture groups containing PANC-1 TSs and NK-92 cells, despite their negligible levels in both the TS and NK single cultures. These results indicated a specific interaction between TSs and NK cells, although it could not be determined which cell type(s) were responsible for the induced secretion. IL-10 and RANTES (regulated upon activation, normal T cells expressed and presumably secreted) were detected in the NK-only group and showed significantly increased levels in the coculture groups containing PANC-1 TSs and NK-92 cells. Significant RANTES expression was also detected under PANC-1 TSs and aPSCs coculture conditions, whereas negligible secretion was observed in either single cell groups. These results provide evidence of the cellular interaction (via cytokine production) under the TIME-on-Chip culture conditions, which supports the validity of this new in vitro 3D model for studying the anticancer activity of NK-92 cells and the effect of aPSCs.

### Phenotypic changes in PSCs under PANC-1 TSs and NK cells coculture conditions

**Fig. 4:**
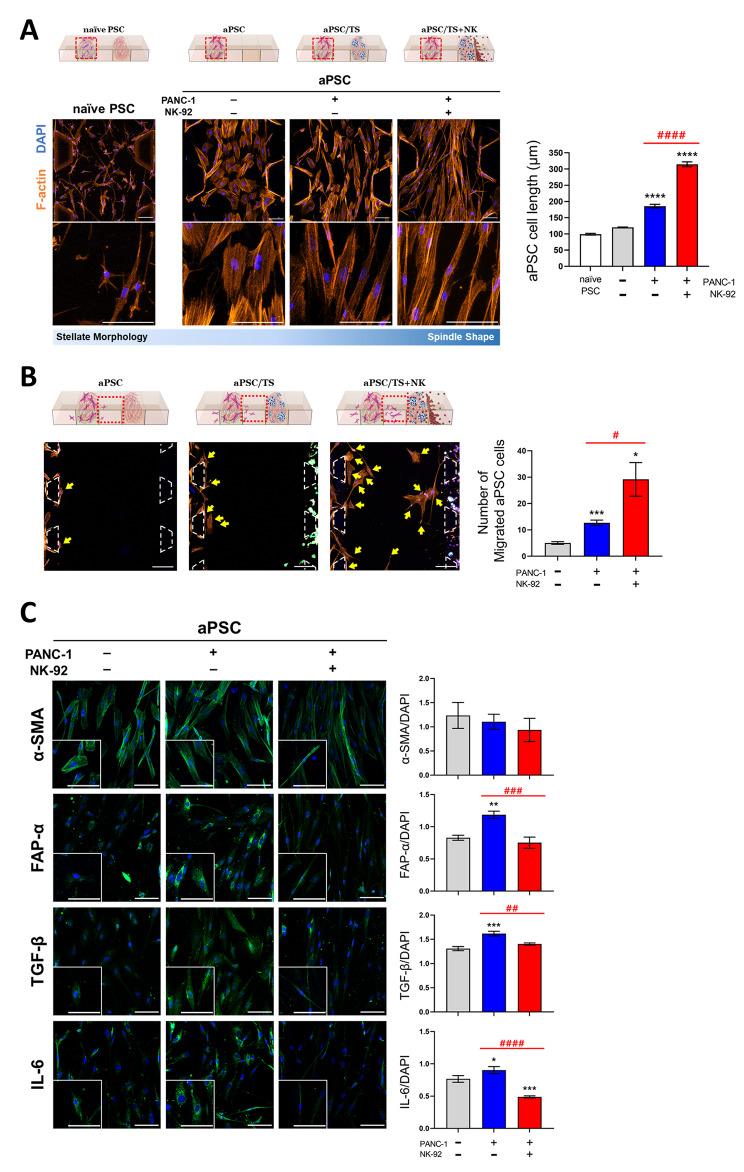
Changes in aPSCs activity under 3D coculture conditions with PANC-1 TSs and NK-92 cells. (**A**) Changes in cell morphology of PSCs when cocultured with PANC-1 TS and NK-92 cells. Orange: F-actin, Blue: DAPI. (**B**) Changes in the migratory ability of aPSCs when cocultured with PANC-1 TS and NK-92 cells, as determined by the number of cells that migrated from the cell channel to the media channel (yellow arrows). DAPI signals were used to count the number of migrating aPSCs. (**C**) The expression of PSC activity markers (green) in aPSCs cocultured with PANC-1 TSs and NK-92 cells. Blue: DAPI. Scale bars: 100 μm (A, C), 200 μm (B). Data represent mean (± SD) values of three independent experiments. *p < 0.05, **p < 0.01, ***p < 0.005, ****p < 0.0001 compared with the aPSCs-only group. #*p* < 0.05, ##*p* < 0.01, ###*p* < 0.005, ####*p* < 0.0001 compared with aPSCs cocultured with PANC-1 TS or with PANC-1 TS and NK-92 cells

To validate the mutual interaction between tumor cells, NK cells, and PSCs in the microchannel chips, phenotypic changes in PSCs observed with 3D coculture conditions were examined. Overall, when cultured in a 3D collagen matrix, aPSCs showed a flat shape with a distinct structure of actin stress fibers instead of the stellate morphology of naïve PSCs (Fig. 4A). Additional alterations were observed when coculturing with PANC-1 TSs, resulting in a 1.5-fold increase in mean length. Notably, the changes were even more pronounced in response to triple coculture involving PANC-1 TSs and NK-92 cells, exhibiting a 2.6-fold increase in mean length compared to the single culture of aPSCs (120 vs. 185 vs. 314 μm). A difference of 1.7-fold increase in cell length was noted when comparing aPSCs cocultured with PANC-1 TSs with or without NK-92 cells (Fig. 4A). The number of aPSCs migrating out of the channel significantly increased by 2.5-fold under coculture conditions involving TSs and by 5.8-fold under triple coculture conditions (cell number: 5 vs. 12 vs. 29). For the triple coculture group, a 2.3-fold increase in aPSCs migration was observed when compared to the PANC-1 TS coculture group (Fig. 4B). On the contrary, no changes were observed in α-SMA levels under coculture conditions involving PANC-1 TS with or without NK-92 cells (Fig. 4C). For FAP-α, TGF-β (transforming growth factor-β), and IL-6, the levels increased under coculture conditions involving PANC-1 TS and reduced when NK-92 cells were added. These results indicated that the coculture with NK-92 cells substantially stimulated the mobility of aPSCs while reducing the myofibroblast activity of aPSCs, suggesting a complex interaction between aPSCs and NK-92 cells via soluble factors in the 3D coculture model.

### Effect of aPSCs on NK cell-mediated cytotoxicity

**Fig. 5:**
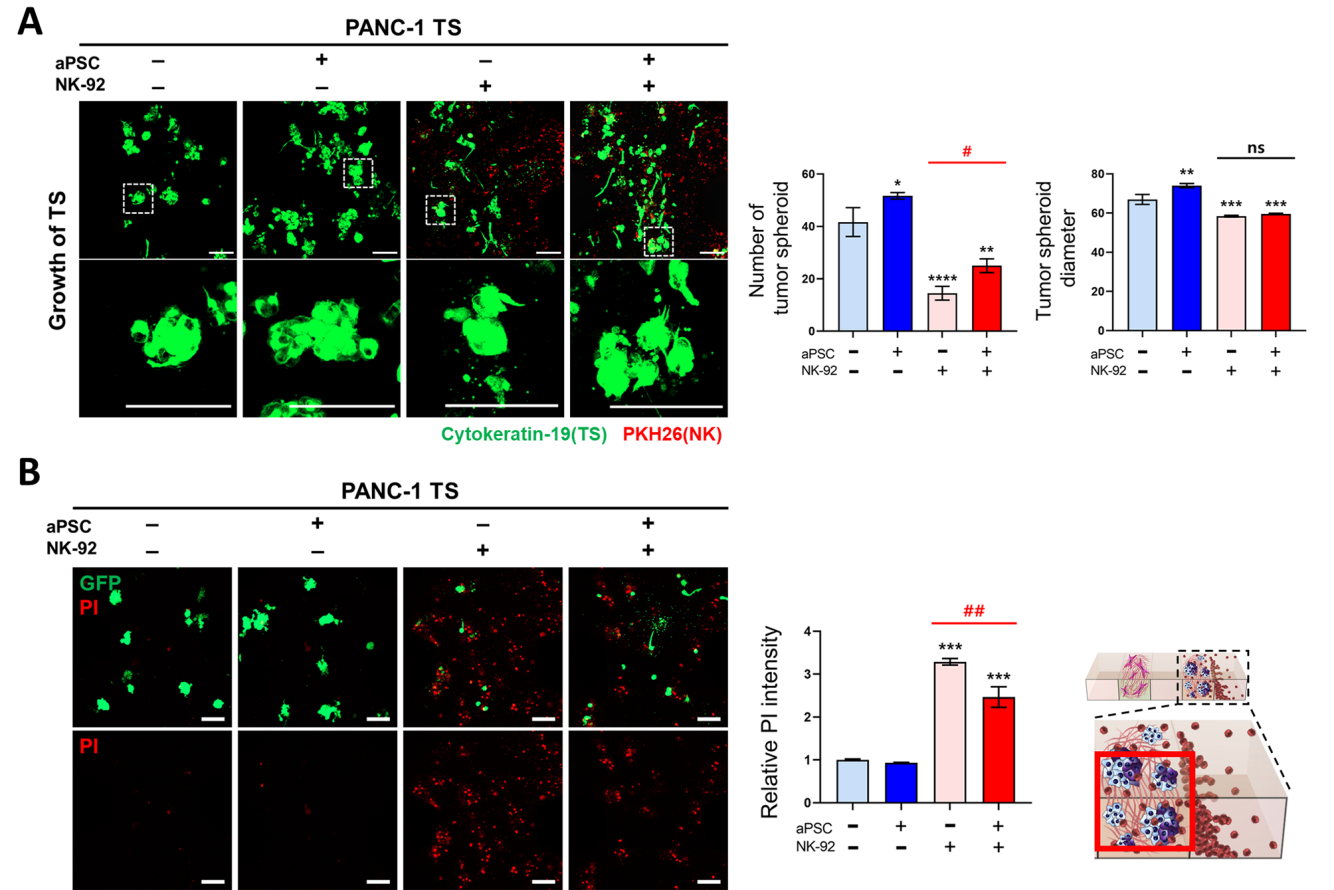
Effect of aPSCs on NK cell-mediated cytotoxicity. (**A**) Effect of aPSCs on NK cell-mediated tumor growth inhibition. Cell aggregates with a diameter larger than 40 μm were considered TS and included in the analysis. PANC-1 TS: cytokeratin-19 (green); NK-92 cell: PKH26 tracker (red). (**B**) Levels of cancer cell death induced by NK-92 cells. Propidium iodide (PI) intensity (red) represent the levels of NK cell-mediated cell death among different groups where live PANC-1 TSs were visualized by the GFP signal (green). *p < 0.05, **p < 0.01, ***p < 0.005, and ****p < 0.0001 compared with the TS-only group. #*p* < 0.05 and ##*p* < 0.01 compared with the TS-NK cell coculture groups with or without aPSCs. ns: not significant. Scale bar: 100 μm. Data represent mean (± SD) values of three independent experiments

To investigate the effect of CAF on the anticancer activity of NK cells, the inhibition of TS growth and cell death mediated by NK-92 cells were compared in PANC-1 TSs cocultured with or without aPSCs. PANC-1 TSs were grown for three days before the loading of NK-92 cells, and the number and size of PANC-1 TSs, as well as the frequency of dead cells was determined after two days of NK cell exposure. Significant growth inhibition of TSs was observed along with a 3-fold increase in the PI signal corresponding to dead cells upon the addition of NK-92 cells (Fig. 5). In coculture settings with aPSCs, the count of remaining TSs following exposure to NK cells exhibited a 72% increase (from 14.5 to 25 according to TSs positive for cytokeratin-19). Conversely, tumor cell killing decreased by 25% (from 3.3 to 2.4 based on relative PI intensity). These outcomes indicate that aPSCs foster cancer cell growth and restrain NK-mediated tumor cell elimination. Nevertheless, the PI signal measured in this experiment may not have originated exclusively from cancer cells, as signals from deceased NK cells could also have contributed to the measured PI signal.

**Fig. 6:**
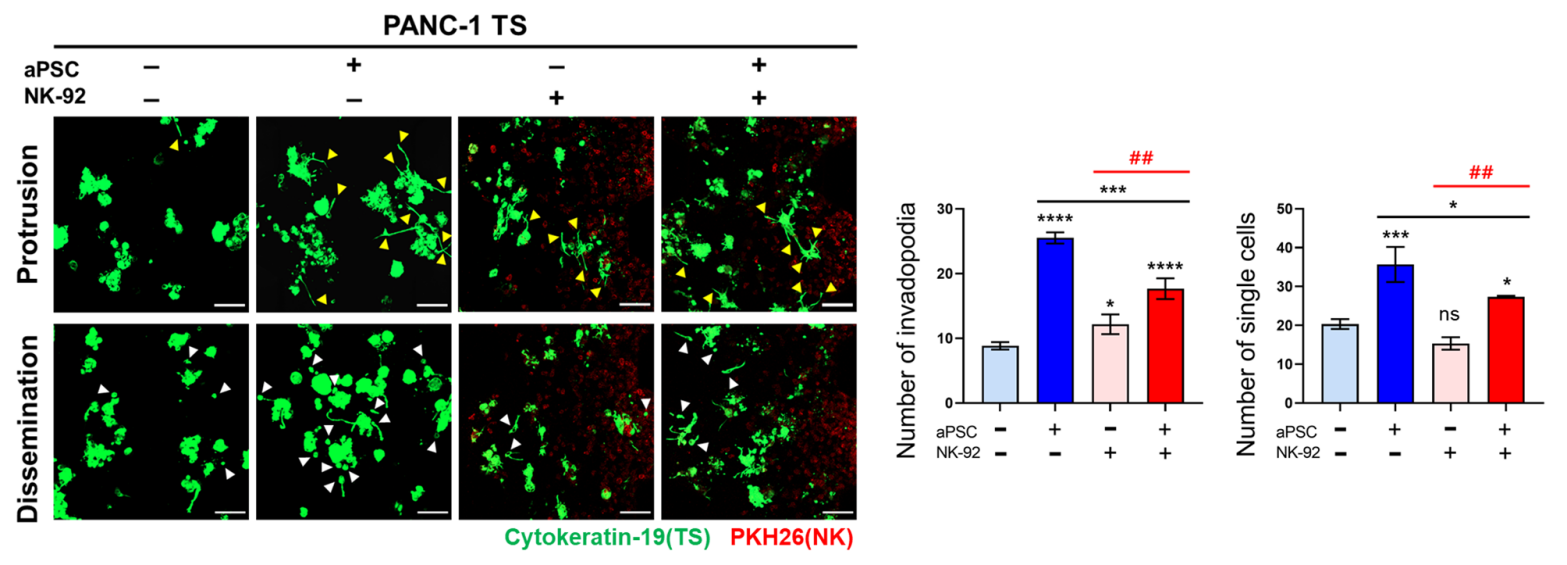
The NK cell-mediated suppression of cancer invasion. Changes in invasive morphology were analyzed using membrane protrusion and single cell dissemination in PANC-1 TSs. Yellow and white arrowheads indicate invadopodia and single cells found apart from cell aggregates, respectively. Cells with a diameter smaller than 20 μm were counted as single cells. *p < 0.05, **p < 0.01, ***p < 0.005, and ****p < 0.0001 compared with the TS-only group. ##*p* < 0.01 compared with the TS-NK cell coculture groups with or without aPSCs. PANC-1 TS: cytokeratin-19 (green); NK-92 cell: PKH26 tracker (red). Scale bar: 100 μm. Data represent mean (± SD) values of three independent experiments

Tumor invasion significantly increased under the influence of aPSCs when evaluated based on the formation of membrane protrusions and the number of single cells disseminated in the ECM (Fig. 6). NK-92 cells demonstrated antiinvasive activity, as shown by a significant decrease in the number of invadopodia (25 vs. 17) and disseminated single cells (36 vs. 27). The number of invadopodia increased paradoxically in PANC-1 TSs exposed to NK-92 cells. This change seemed transitional since no changes were observed in single-cell dissemination that typically follows the invadopodia formation during cancer cell invasion. Of note, the antiinvasion effect of NK-92 cells was observed only in TSs under coculture conditions involving aPSCs and not in the absence of aPSCs. These findings suggest two potential scenarios: either the antiinvasive activity of NK cells was enhanced under coculture conditions with aPSCs, or the protumorigenic activity of aPSCs was suppressed by NK-92 cells. The latter scenario appears more likely, supported by the decreased expression of aPSC activity markers observed in coculture with NK-92 cells (Fig. 4C).

### Effect of aPSCs on NK infiltration and ECM remodeling

**Fig. 7:**
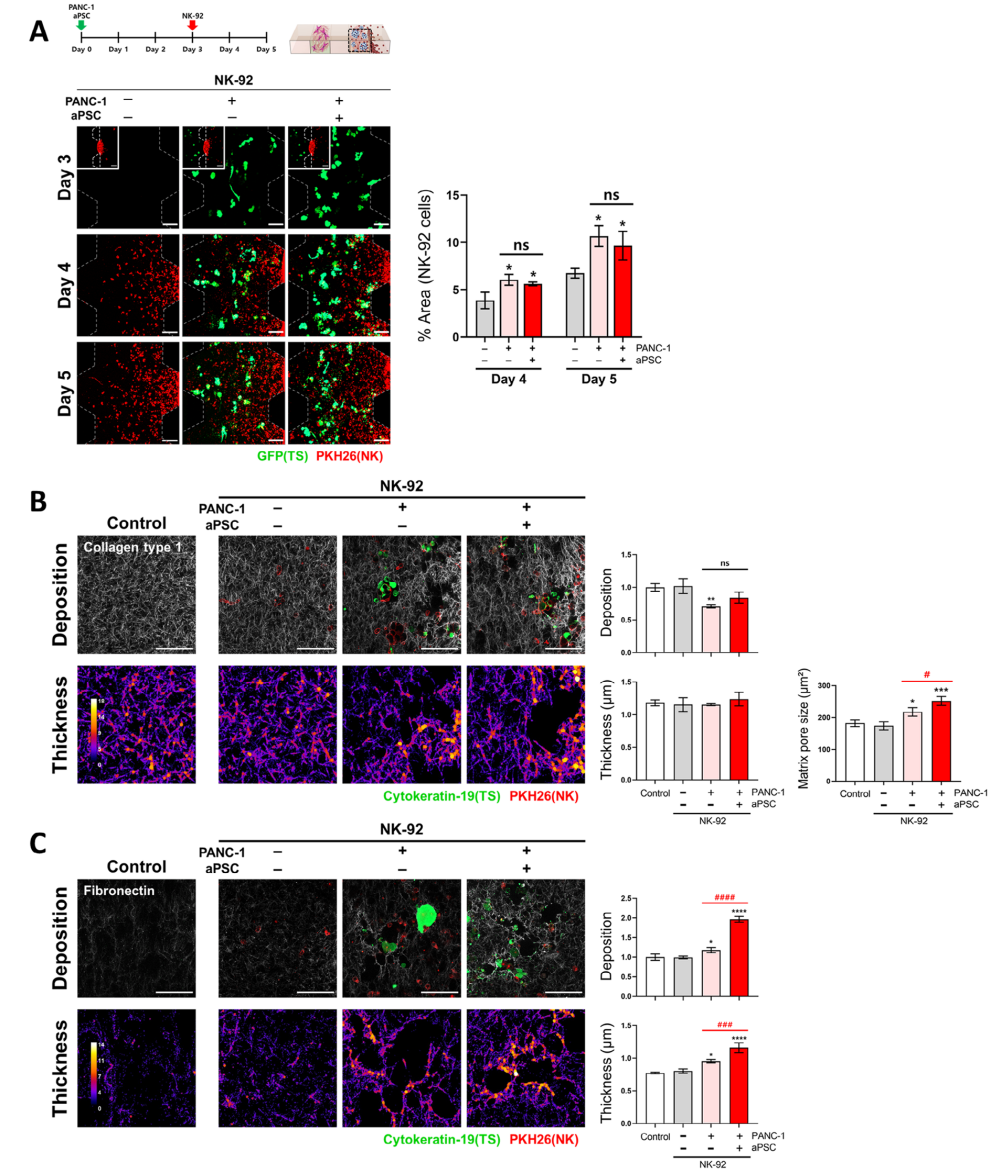
Effects of aPSCs coculture on NK-92 cell infiltration and extracellular matrix (ECM) organization. (**A**) Infiltration of NK-92 cells into matrix-only channels and into tumor channels in the presence or absence of aPSCs. (**B**) Changes in collagen type 1 matrix structure with respect to deposition level, fiber thickness, and matrix pore size. (**C**) Changes in fibronectin matrix structure with respect to deposition level and fiber thickness. Changes in matrix structure were compared when NK-92 cells were allowed to infiltrate into the matrix-only channel and into tumor channels in the presence or absence of aPSCs. For the analysis of ECM fiber thickness, refer to the method section. *p < 0.05, **p < 0.01, ***p < 0.005, and ****p < 0.0001 compared with the NK cells only group. #*p* < 0.05, ###*p* < 0.005, ####*p* < 0.0001 compared with the TS-NK cell coculture groups with or without aPSCs. ns: not significant. A control group with cell-free matrix is shown for reference. PANC-1 TS: GFP or cytokeratin-19 (green); NK-92 cell: PKH26 tracker (red). Data represent mean (± SD) values of three independent experiments. Scale bar: 100 μm

To determine whether CAF-induced suppression of the anticancer activity of NK cells could be attributed to changes in the infiltration ability of NK cells, the distribution of NK-92 cells in TS channels was examined. Infiltration of NK-92 cells increased under TS coculture conditions on days 4 and 5; however, no changes were observed when aPSCs were added to the culture (Fig. 7A). ECM remodeling was also evaluated concerning the density and fibrillar structure of collagen type I and fibronectin. The density of the collagen matrix decreased under TSs coculture conditions, which may be due to degradation of the collagen matrix by tumor cells (Fig. 7B). Moreover, the pore size of the collagen matrix increased, which seemed to be associated with increased infiltration of NK-92 cells (Fig. 7A). The degree of collagen degradation remained unchanged; however, a further increase in the pore size of the collagen matrix was observed under the triple coculture conditions (Fig. 7B). This result suggests that NK cells migration may be facilitated by the size of the matrix pores only to a certain degree; indeed, increased pore size above 217 µm^2^ under aPSCs coculture may not promote the migration of NK-92 cells (227 µm^2^ cross-sectional size). In contrast to collagen, fibronectin deposition and fiber thickness significantly increased upon tumor cells and aPSCs coculture, indicating no apparent correlation with NK-92 cell infiltration (Fig. 7C).

### Effect of aPSCs on the expression of NK activation markers

**Fig. 8:**
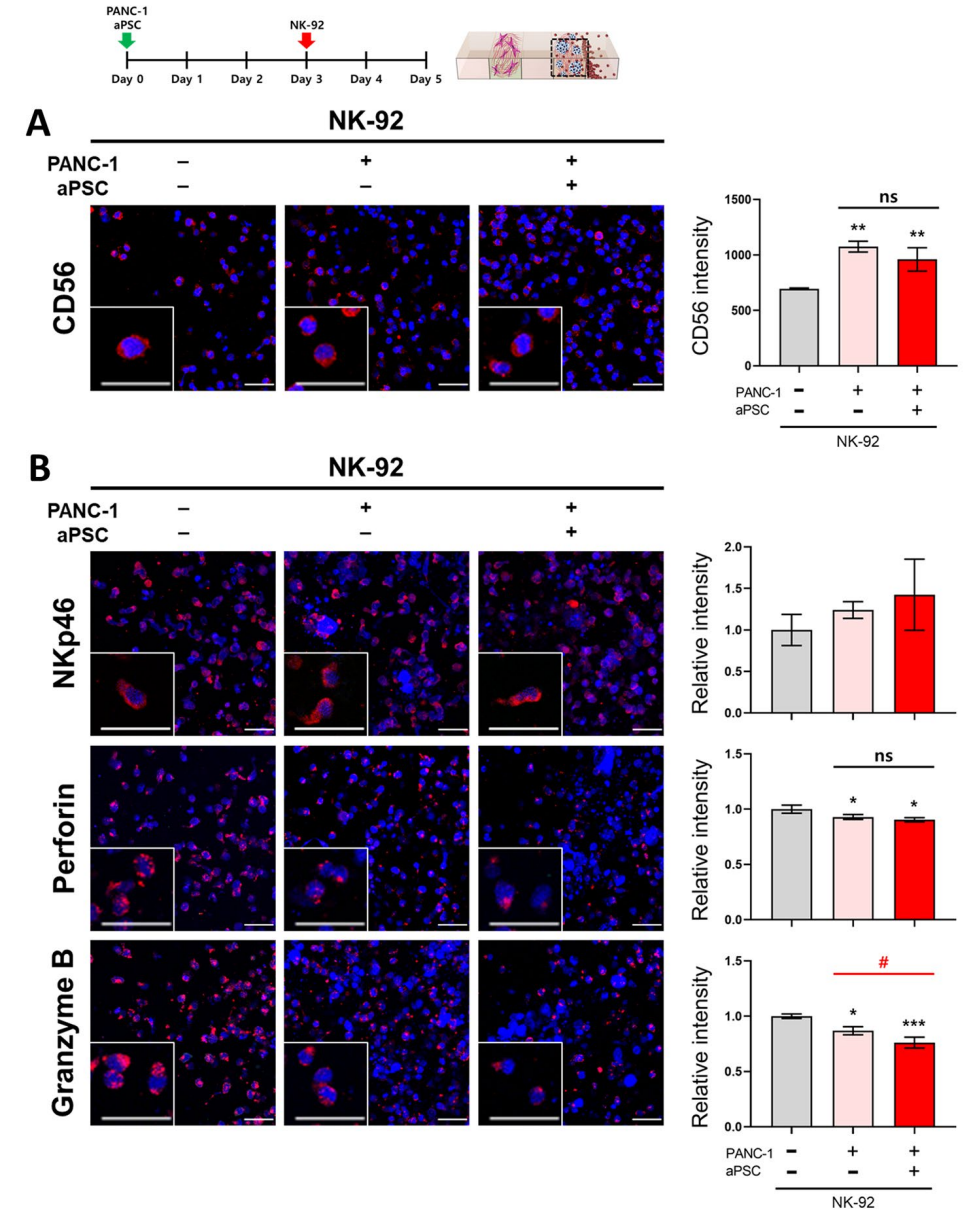
Effect of aPSCs on NK cell activation. Changes in the expression of (**A**) CD56, a phenotypic marker of NK cells, and (**B**) activity markers, namely NKp46, perforin, and granzyme B, in NK cells when cultured alone or in coculture with PANC-1 TS alone or PANC-1 TS and aPSCs. The relative intensity of NK activity marker expression was calculated using the relative CD56 intensity. NK cell markers (red); nuclei of tumor and NK cells (DAPI, blue). Scale bar: 50 μm. Data represent mean (± SD) values of three independent experiments. *p < 0.05, **p < 0.01, ***p < 0.005 compared with the NK-only group. #*p* < 0.05 compared with NK-92 cells cocultured with PANC-1 TSs alone or PANC-1 TSs and aPSCs. ns: not significant

To determine the potential mechanism of the CAF-mediated downregulation of NK cell cytotoxicity, changes in the expression levels of activating receptors and cytolytic granules were determined under different culture conditions. The intensity of CD56 expression was used to represent the distribution of NK-92 cells in the channel, since CD56 is a phenotypically expressed marker of NK-92 cells [31]. The CD56-positive cell population in the tumor channel increased 1.5-fold under coculture conditions with PANC-1 TS, either with or without aPSCs (Fig. 8A), which was consistent with the data obtained using the PKH26 tracker (Fig. 7A). The expression of three activity markers of NK cells, namely NKp46, perforin, and granzyme B, was evaluated after normalization to relative CD56 intensity. No differences in the levels of NKp46 were observed among the three experimental groups (Fig. 8B), whereas those of perforin, a secretory granule of NK cells, were significantly decreased in the presence of PANC-1 TSs, indicating the exhaustion of NK cell activity; however, no effect was observed with the addition of aPSCs to the culture. Granzyme B expression was significantly decrease upon PANC-1 TSs culture and a further reduction was observed under coculture conditions with aPSCs (Fig. 8B), indicating that the aPSC-mediated suppression of the cytotoxic activity of NK-92 cells may be attributed to the downregulation of granzyme B secretion.

## Discussion

3D in vitro tumor models comprising tumor and stromal cells embedded in an ECM have been considered the most adequate mimic of in vivo tumors and thereby representing the best conditions for studying the changes in the anticancer activity of cells within the TIME. However, mechanistic studies of the interactions between NK and cancer cells under an immune-suppressing microenvironment have been limited due to the lack of a pathophysiologically relevant in vitro tumor model. A microfluidic chip-based 3D tumor model provides several advantages, such as less sample use, shorter experimental time, adaptability for microscopic imaging, and, more importantly, flexible compartmental design to accommodate coculture conditions for various cell types of interest [32]. Indeed, a microfluidic culture platform was previously used to show that NK cell penetration through cell-cell junctions is significantly faster than that of antibodies in tumor spheroids [27] and that compromised NK cell surveillance is due to NK cell exhaustion via overexpression of cytotoxic T-lymphocyte-associated protein-4 [26]. The TIME-on-Chip model of human pancreatic tumors herein presented consists of a collagen-based 3D culture of pancreatic TSs, stellate cells, and NK cells using microfluidic chips and is suitable for evaluating the crosstalk between cell types via soluble factors and ensuing phenotypic changes. Overall, this in vitro culture setting allowed the successful presentation of the infiltrating mobility profile of NK cells, changes in cells secretome, phenotypic changes in aPSCs induced by NK-92 cells, and the suppression of NK cell-mediated cytotoxicity by aPSCs.

The complex network of cells that constitute the PDAC TIME includes mutual interactions via low-molecular weight signaling molecules and cytokines that promote tumor initiation, progression, and metastasis [33]. In this study, MCP-1 was significantly elevated in the tumor-only group, and a similar level was observed in the coculture groups containing TSs, indicating that tumor cells are a major source of MCP-1 in our PDAC culture model. MCP-1 is secreted by cancer and stromal cells to support tumor growth, progression, and metastasis [34]. Other protumorigenic cytokines, such as GROα/β/γ, IL-8, and IL-6, are known to be secreted by cancer and stromal cells [35–39], whereas IL-2 and IL-3 are secreted from activated T cells to regulate the activity and maturation of immune cells to be able to eliminate tumor cells [40–43]. IL-10 and RANTES are known to be secreted by cancer and stromal cells but also by immune cells; therefore, experimental data on their role on tumor growth remain controversial [44–47]. Overall, the present findings suggest that the anticancer effect undergoing in the PDAC TIME network may result from the activity of different cytokines with pro- or antitumor functions. This also emphasizes the usefulness of our coculture model for further studies on the detailed underlying mechanisms of cytokine secretion and their contributions to the anticancer effects of NK cells.

Lymphoid cells, including NK cells, are known to migrate along pre-existing fibrillar strands in a 3D collagen matrix using ‘amoeboid migration’, inducing minimal structural changes on the matrix [48, 49]. This type of migration can be particularly effective in tissues but has been shown to be dependent on ECM density. Collagen density affects various physical properties of collagen gels, such as pore size, stiffness, and cell migration [50], with varying concentrations of collagen, ranging within 1.2–4 mg/mL, being used in collagen-based 3D tumor models [27, 51, 52]. This study tested three different collagen concentrations (1, 1.2, and 1.5 mg/mL) to demonstrate the concentration-dependent changes in the matrix pore size and concomitant impact on NK-92 cells penetration. Noteworthily, NK-92 cell infiltration increased by more than 1.3-fold in the presence of PANC-1 TSs regardless of the collagen concentration used compared with that in cell-free matrix conditions, suggesting chemotaxis by tumors. MCP-1 plays an important role in immune responses by regulating the infiltration and migration of various immune cells, including NK cells [53]. Herein, MCP-1 was found to be significantly expressed in the conditioned media of coculture groups containing PANC-1 TSs; hence, MCP-1 may be responsible for the chemotactic migration of NK-92 cells in the presence of the PANC-1 TSs. Nonetheless, the involvement of other chemotactic factors cannot be excluded.

Reduced antitumor activity of NK cells may be attributed to insufficient NK cell infiltration and NK cell dysfunction in response to immune suppressive effects of tumor microenvironments, among which CAFs having various roles in tumor progression are considered the most important [54]. CAFs play a major role in ECM remodeling not only via direct ECM protein deposition and restructuring but also by stimulating tumor cells for ECM remodeling in an indirect mode [55, 56]. The present study also demonstrated the suppressed antitumor effect of NK-92 cells against PANC-1 TSs in the presence of aPSCs. Although aPSCs in the present study were cultured in a separate microchannel to interact with tumor cells only via soluble factors, a myofibroblastic phenotype was evident, characterized by 2 to 5-fold higher expression of α-SMA and FAP-α compared to naïve PSCs (data not shown) [57]. Under indirect coculture conditions with these aPSCs, ECM remodeling was induced around PANC-1 TS as shown by increased fibronectin deposition in the tumor channel. However, the overall distribution of NK cells was maintained, which may be attributed to amoeboidal-mesenchymal movement of NK cells [58]. In contrast, PDAC patient-derived aPSCs showed reduced cytotoxic activity of NK cells, as evidenced by the lower expression of activity markers, such as interferon-γ, granzyme B, and CD107a [20]. Colorectal and endometrial cancer-derived fibroblasts were reported to inhibit NK cell activity via the downregulation of NK cell activation receptors (CD69, NKG2D, DNAM-1, NKp30, NKp44, and CD27) and cytolytic granules (perforin and granzyme B), suggesting that this characteristic of CAFs is not limited to pancreatic carcinoma [59–61]. Although little is known about the precise mechanism by which CAFs control NK cell activity, cytokines secreted by CAFs and cancer cells, such as TGF-β, prostaglandin E2, indoleamine-2,3-dioxygenase, and IL-6, were reported to regulate NK cell activity [11, 16, 62]. Furthermore, tumor cell-derived IL-6 and IL-8 activate molecular signals in NK cells that reduce the expression of activation markers (NKp30, NKG2D, and granzyme B), thereby impairing NK cell function [63]. Since significant IL-6 and IL-8 levels were detected in the conditioned media of aPSCs, a similar molecular mechanism may contribute to granzyme B reduction in NK-92 cells, which warrants further evaluation.

Despite the successful demonstration of the reduced antitumor activity of NK cells under the influence of aPSCs using a 3D TIME-on-chip model, the current study has limitations, including but not limited to the following points. Firstly, our TIME-on-chip model was tailored with specific dimensions and optimized culture conditions to ensure appropriate growth of each cell type, their interaction via soluble factors, and subsequent immunostaining visualization. However, these conditions might not entirely mirror the in vivo tumor environment, potentially imposing limitations on the interpretation and applicability of our study findings. Secondly, the use of specific cells, such as PANC-1 cells, PSC (HpaSteC), and NK-92 cells could potentially limit the reliability and representativeness of the model. Additional studies using this model with other cell types, such as other cancer cells, PSCs and NK cells of different origins, and patient-derived primary cells commonly used in many studies, can provide additional clinical insights concerning crosstalk between tumor and NK cells or CAFs and NK cells. Third, dysregulation of NK cell activity may be attributed to various factors in the tumor microenvironment that were not included in our model. In addition to aPSCs acting as immunosuppressive CAFs, the inclusion of regulatory T cells, myeloid-derived suppressor cells, tumor-associated neutrophils, or tumor-associated macrophages, which are known to exhibit immunosuppressive activity against NK cells, could potentially influence the outcomes, introducing varied implications [54]. Finally, this study focused on the establishment of a TIME-on-chip model for evaluating NK cell infiltration and tumor cell death under the influence of CAFs, rather than elucidating the detailed mechanism of NK cell suppression. Further mechanistic studies using specific inhibitors or restorative agents as putative targets may lead to the discovery of novel NK cell-based treatment strategies.

## Conclusions

In summary, a microfluidic channel chip-based PDAC tumor model was established in which PANC-1 TSs, aPSCs, and NK-92 cells were cocultured to allow mutual interaction via soluble factors, as well as direct contact between TSs and NK cells in a 3D environment. Hence, this represents a novel TIME-on-chip model for studying NK cell-mediated anticancer effects. The anticancer efficacy of NK-92 cells was suppressed in the presence of aPSCs, which was attributed to the dysregulation of the cytotoxic activity of NK cells, with no changes in the infiltration profile of NK cells in the tumor parenchyma. Overall, the TIME-on-chip model can be useful for mechanistic studies of NK-mediated anticancer activity and for screening NK-based immunotherapies in vitro.

### Electronic supplementary material

Below is the link to the electronic supplementary material.

**Additional file 1: Fig. S1**. Comparison of matrix pore size in the cell-free matrix and PANC-1 TS channels according to collagen concentration. An increase in pore size was observed in the presence of PANC-1 TS. Collagen type 1 fibers (white) were overlaid with PANC-1 TS (GFP, green). Scale bar: 100 μm. A minimum of 10 regions of interest were selected from three fields obtained from each microchannel chip. Data represent mean (± SD) values of three independent experiments. *p < 0.05, ****p < 0.0001.

**Additional file 2: Fig. S2**. Secretome analysis of conditioned media derived from microchannel cultures of PANC-1 TS, aPSCs, and NK-92 cells, alone or under coculture conditions. (**A**) Only 10 proteins showed significant levels. (**B**) Relative expression of 10 factors identified in the culture medium of PANC-1 TS, aPSCs, and NK-92 cells. Several factors, including IL-6, were specifically induced by PANC-1 TS–NK-92 cell interactions.

## Data Availability

All data generated or analyzed during this study are included in this published article and its supplementary information files.

## List of abbreviations

3D: three-dimensional
aPSC: activated pancreatic stellate cell
CAF: cancer-associated fibroblast
ECM: extracellular matrix
G-CSF: granulocyte-colony stimulating factor
GFP: green fluorescent protein
IL: interleukin
MCP: monocyte chemoattractant protein
NK: natural killer
PDAC: pancreatic ductal adenocarcinoma
PSC: pancreatic stellate cell
RANTES: regulated upon activation, normal T cell expressed and presumably secreted
TGF: transforming growth factor
TIME: Tumor immune microenvironment
TS: tumor spheroid
