# Supplementary figures and images for: Suppression of the antitumoral activity of natural killer cells under indirect coculture with cancer-associated fibroblasts in a pancreatic TIME-on-chip model
Source: Cancer Cell Int. 2023 Sep 27;23:219. doi: 10.1186/s12935-023-03064-9 (PMC10536815; doi:10.1186/s12935-023-03064-9)

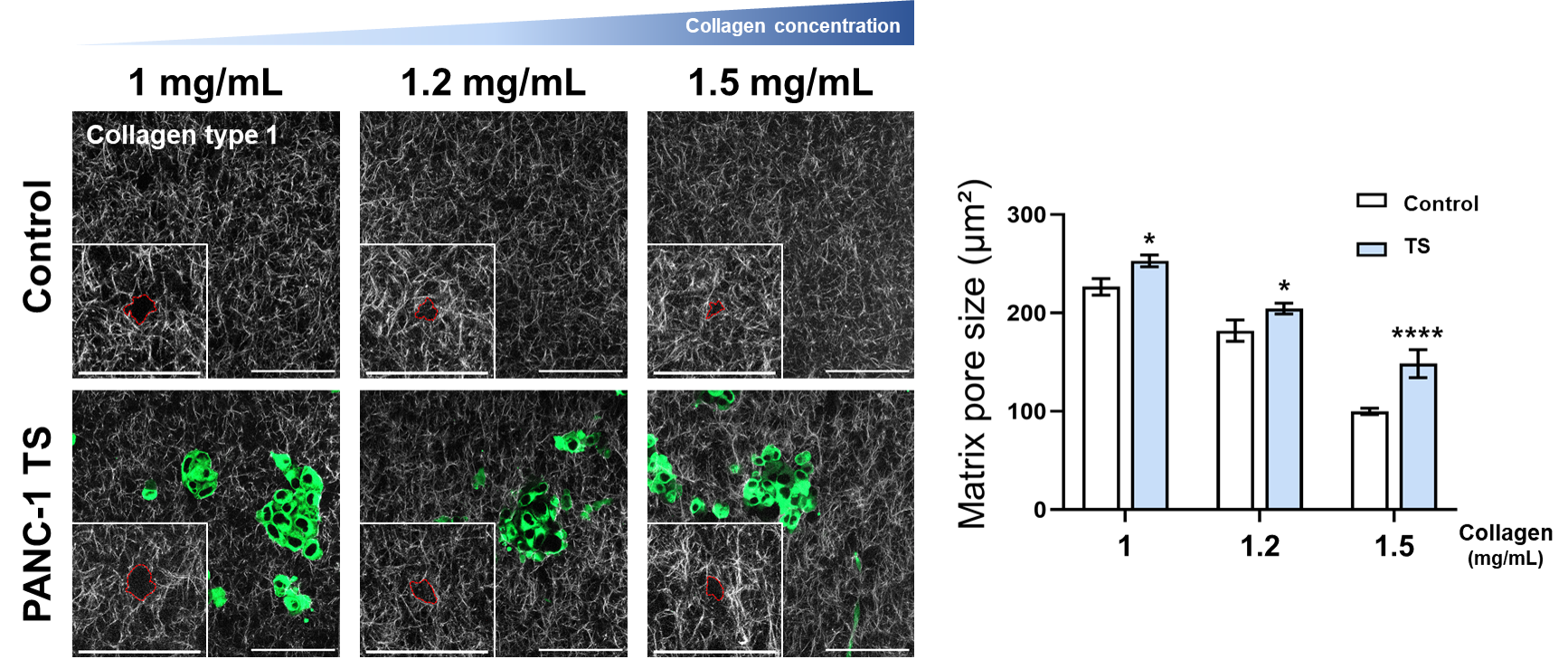

Supplement: Supplementary file 1 — Additional file 1: Fig. S1. Comparison of matrix pore size in the cell-free matrix and PANC-1 TS channels according to collagen concentration. An increase in pore size was observed in the presence of PANC-1 TS. Collagen type 1 fibers (white) were overlaid with PANC-1 TS (GFP, green). Scale bar: 100 μm. A minimum of 10 regions of interest were selected from three fields obtained from each microchannel chip. Data represent mean (± SD) values of three independent experiments. *p < 0.05, ****p < 0.0001. [file 12935_2023_3064_MOESM1_ESM.tif]

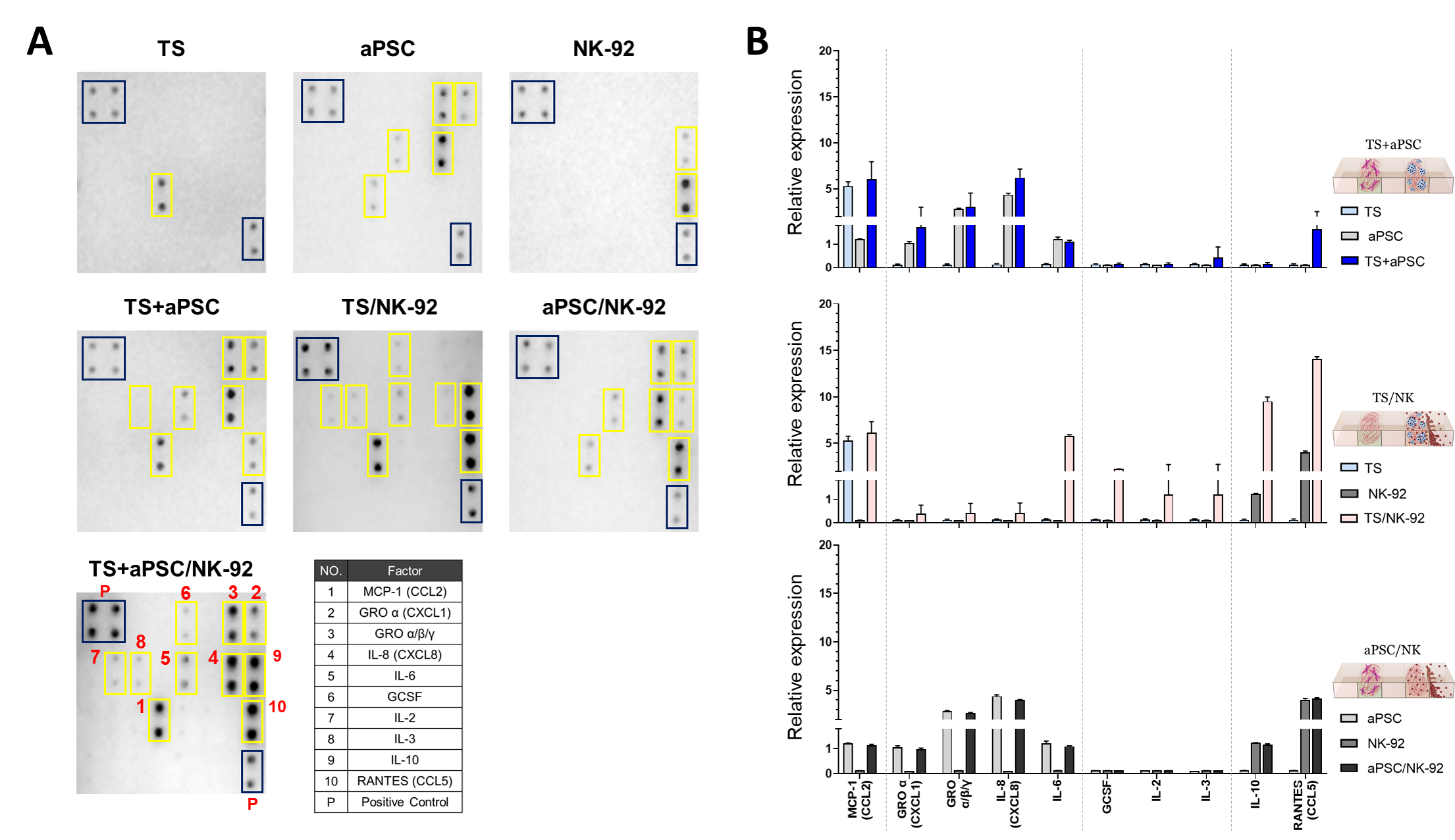

Supplement: Supplementary file 2 — Additional file 2: Fig. S2. Secretome analysis of conditioned media derived from microchannel cultures of PANC-1 TS, aPSCs, and NK-92 cells, alone or under coculture conditions. (A) Only 10 proteins showed significant levels. (B) Relative expression of 10 factors identified in the culture medium of PANC-1 TS, aPSCs, and NK-92 cells. Several factors, including IL-6, were specifically induced by PANC-1 TS–NK-92 cell interactions. [file 12935_2023_3064_MOESM2_ESM.tif]
